# STAT3 inhibition in combination with CD47 blockade inhibits osteosarcoma lung metastasis

**DOI:** 10.3389/fimmu.2025.1608375

**Published:** 2025-06-03

**Authors:** Pradeep Shrestha, Rejeena Shrestha, You Zhou, Rafal Zielinski, Waldemar Priebe, Eugenie S. Kleinerman

**Affiliations:** ^1^ Department of Pediatrics-Research, The University of Texas MD Anderson Cancer Center, Houston, TX, United States; ^2^ Institute for Cell Therapy Discovery and Innovation, The University of Texas MD Anderson Cancer Center, Houston, TX, United States; ^3^ Department of Experimental Therapeutics, The University of Texas MD Anderson Cancer Center, Houston, TX, United States; ^4^ Department of Cancer Biology, The University of Texas MD Anderson Cancer Center, Houston, TX, United States

**Keywords:** osteosarcoma lung metastasis, STAT3, CD47-SIRPα, anti-tumor immunity, WP1066

## Abstract

**Background:**

New therapies are urgently needed for patients with osteosarcoma (OS). STAT3 and CD47 are potential therapeutic target in OS. Here we investigated the therapeutic activity of the orally bioavailable STAT3 inhibitor, WP1066, and anti-CD47 antibody using OS mouse models.

**Methods:**

Cytotoxic effect of WP1066 against OS cell lines and its immunomodulatory effects were evaluated *in vitro*. Experimental metastasis and orthotopic syngeneic mouse models were used to investigate the therapeutic efficacy of WP1066 and anti-CD47 antibody. Further flow cytometric analysis was performed.

**Results:**

STAT3 was constitutively activated in multiple human and mouse OS cell lines. WP1066 suppressed STAT3 activation and induced apoptosis. WP1066 reduced the viability and proliferation of MDSCs and increased the expression level of MHC-II, and CD80 in macrophages. We demonstrated that WP1066 monotherapy prolonged the survival of mice with OS lung metastasis using an experimental metastasis and an orthotopic model. The therapeutic effect was significantly increased when WP1066 was combined with anti-CD47. This was associated with increased frequency of activated CD8^+^ T cells, NK cells and macrophages in the lungs and LDLNs.

**Conclusion:**

Our preclinical studies support further investigation of targeting STAT3 and CD47 as novel immunotherapeutic approach against OS lung metastasis.

## Background

Osteosarcoma (OS) is a highly aggressive malignant bone tumor, most common in children and young adults. Approximately 20% of newly diagnosed patients present with metastatic disease primarily in the lungs. With significant advances in diagnosis and aggressive chemotherapy the 5- year survival rate for patients with localized disease is approximately 60% but only 20% for patients with metastases or recurrent disease (1, 2). Because of the poor outcome of the patients with metastatic OS, which has not changed for decades, novel therapeutic approaches are critically needed to improve the outcomes (1–3).

Signal transducer and activator of transcription 3 (STAT3) is a member of STAT family that becomes activated in response to various growth factors and cytokines (4). Activated STAT3 (phosphoSTAT3^Y705^) functions as a transcription factor and regulates expression of multiple genes and plays critical role in fundamental processes including proliferation, differentiation, and survival (5, 6). While activation of STAT3 is tightly regulated in non-malignant cells, it is persistently activated in most hematologic malignancies and solid cancers, including osteosarcoma (5, 7, 8). STAT3 activation is critical for survival and proliferation of osteosarcoma (8–10). In fact, overexpression of STAT3 is correlated with poor prognosis of multiple cancers along with osteosarcoma (11). Furthermore, what makes STAT3 a promising target is that it plays a crucial role in tumor-stromal cells including immune cells in maintaining an immune-suppressive tumor immune microenvironment (TME) to support and promote tumor progression (4, 12–16).

Liposomal muramyl tripeptide-phosphatidyl ethanolamine (L-MTP-PE) is a muramyl dipeptide analog that activates macrophages through interaction with nucleotide-binding oligomerization domain containing protein 2 (NOD2) and promotes tumoricidal activity of macrophages (17). The liposomal formulation promotes phagocytosis by macrophages. When administered intravenously these liposomes are taken up in the liver, spleen and lungs. Activation of pulmonary macrophages activation by L-MTP-PE immunotherapy improved event-free and overall survival in patients with newly diagnosed and metastatic OS (18–20). This therapeutic success supports the concept of identifying agents that enhance macrophage functions such as phagocytosis. CD47, a glycoprotein also called an innate immune checkpoint, is a ligand to signal receptor protein α (SIRPα) expressed on innate immune cells including macrophages. Binding to SIRPα delivers “don’t eat me” signal that inhibits phagocytosis, thereby allowing tumor cells to evade immune clearance. A wide range of hematologic and solid tumors-*including OS*- upregulate CD47 as a mechanism of immune evasion. Notably, higher CD47 expression is associated with worse overall survival in OS patients (21–23). Moreover, CD47 expression is upregulated in response to chemotherapy in OS, and this therapy induced increase in CD47 levels has been linked to patient mortality (22). Beyond its role in suppressing phagocytosis, the CD47-SIRPα interaction also regulates the activation of innate immune cells, broadly dampening the anti-tumor immune response. Therapeutic blockade of the CD47-SIRPα axis has been shown to restore macrophage mediated phagocytosis, natural killer (NK) cells mediated cytotoxicity, and facilitate cross presentation by dendritic cells to promote robust T cell responses (24–28).

In this study, we hypothesized that overexpression of phosphoSTAT3^Y705^ and CD47 in OS are therapeutic targets and that blocking these two pathways will synergize and potentiate anti-tumor response. WP1066 (29–31) is an orally bioavailable STAT3 inhibitor that blocks phosphorylation of STAT3 *in vitro* and *in vivo*. WP1066 induces apoptosis *in vitro* as well as anti-tumor efficacy *in vivo* in multiple tumors (32–34). Here, we show that STAT3 inhibition by WP1066 induced cell apoptosis and reduced tumor cell proliferation *in vitro*. Using both syngeneic immune-competent orthotopic and experimental OS lung metastasis models, we demonstrated the *in vivo* therapeutic activity of WP1066 against OS lung metastasis. This efficacy was enhanced by combining WP1066 with CD47 neutralization resulting in a significantly prolonged median survival time.

## Materials and methods

### Cell lines and culture conditions

Human OS cell lines SAOS-2 (HTB-85), 143B (CRL-8303), MG63 (CRL-1427), human fetal osteoblastic cells (hFOB; CRL-11372) and mouse macrophage cell line RAW264.7 (TIB-71) cells were obtained from American Type Culture Collection (ATCC) (35). Human metastatic LM7 and MG63.2 were derived from SAOS-2 and MG63 respectively (35). CCH-OS-O and CCH-OS-D cells were derived from the primary tumor of patients (35). The OS17 cell line (36) was used to generate OS17-GFP cells and provided by Dr. Amer Najjar (MD Anderson). LM7-GFP was generated in our laboratory.

The mouse metastatic OS cell line K7M3 was derived from K7 (37) mouse OS cells (38). Dunn and DLM8 cell lines were obtained from Dr. Asai Tatsuya (39). Luciferase expressing K7M3 cells (K7M3-luc) were kindly provided by Dr. Timothy M. Fan (University of Illinois) originally generated by S.Y. Kim at NCI (40, 41). All cells except hFOB, were cultured and maintained in Dulbecco’s modified Eagle’s medium supplemented with 10% heat-inactivated fetal bovine serum, 1% Penicillin/Streptomycin, 1% L-Glutamine, 1X nonessential amino acids, 1X minimal essential medium vitamin solution. The hFOB cells were maintained in DMEM/F12 (1:1) containing 10% FBS and 300 µg/ml neomycin (G418). All cells were regularly tested for mycoplasma contamination using MycoAlert mycoplasma detection kit (Lonza).

### Animal studies and tumor models

Animal experiments performed in the study were in accordance with approved IACUC protocols. BALB/c female mice (6–8 weeks old) were used in the studies and were purchased from Charles River at NCI Frederick. Each group contained at least 5 mice. For the experimental OS-lung metastasis model, BALB/c mice were injected i.v with 0.25x10^6^ K7M3-luc cells. Animals were randomized after tumor cells injection. Blinding was not done for the *in vivo* experiments. Mice were injected with D-luciferin (GoldBio, 150 mg/kg) for bioluminescence *In vivo* Imaging system (IVIS) imaging to assess tumor burden.

The orthotopic OS model was also employed to assess the therapeutic effect on established lung metastases that arose from the primary tumor (38). Briefly, the right leg of 7–8 weeks old BALB/c mice was dehaired 24 hrs before tumor cell injection. K7M3-luc cells (0.2x10^6^ cells in 10 µl of PBS) were injected into the right tibial medullary cavity using a 27-gauze needle and 100 µl Hamilton syringe to establish an orthotopic primary OS tumor. The right leg bearing primary tumor was amputated at day 20 post intra-tibial tumor cell injection, a time when lung micrometastases are present (38).

### Treatment

WP1066 (29, 42) was administered via oral gavage (50 mg/kg) in vehicle of DMSO/PEG300 (1:4) every day for 5 days a week for three weeks (29, 32, 43). Control cohorts received vehicle only. For anti-CD47 treatment, mice were treated with 400 µg of anti-CD47 antibody (Clone MIAP410; Bioxcell) in 200 µl PBS intra-peritoneally every 48 hrs three doses a week for total of eight doses as shown in experimental schema (44). In combination treatment, WP1066 treatment was initiated earlier than anti-CD47 antibody treatment to achieve stable plasma concentration that requires multiple administrations (29).

### Spheroid model and WP1066 treatment

To generate tumor spheroids, OS17-GFP and LM7-GFP cells (1x10^4^) were seeded in clear, round-bottom 96 well ultra-low attachment plates (Corning) in complete DMEM media. The plates were then centrifuged for 5 mins at 1000 g and incubated at 37°C. After 24 hrs, the spheroids were treated with the indicated concentration of WP1066 or DMSO only as control. Plates were incubated in the IncuCyte S3 live-imaging system (Essen Biosciences) and scanned every 6 hrs for 5 days.

### *In vitro* generation of myeloid derived suppressor cells (MDSCs) and T cell suppression assay

MDSCs were generated from bone marrow isolated from naïve female BALB/c mice as described before (45). Single cells isolated from femur and tibia were plated (5x10^6^ cells/plate) in non-tissue culture treated 10 cm plates in complete RPMI media (basal RPMI with 10% FBS and penicillin and streptomycin) supplemented with recombinant mouse GM-CSF (40 ng/ml; Biolegend) and IL6 (40 ng/ml; Biolegend). After 4 days in culture, Bone marrow derived MDSCs (BM-MDSCs) were harvested and washed with complete RPMI for assays. To determine the effect of WP1066 on BM-MDSCs, BM-MDSCs were replated in 10 cm non-TC plates (5x10^6^ cell) and treated with the indicated concentration of WP1066. After 24 hrs cells were harvested and analyzed for surface markers including CD11b, Ly6G, Ly6C, and Ki67 (proliferation marker) expression by flow cytometer.

For T cell suppression assays (46), splenocytes harvested from naïve BALB/c mice were incubated in tissue culture treated plates for 45 mins to isolate T cells. T cells were labelled with 2.5 µM Cell Trace Carboxyfluorescein succinimidyl ester (CFSE, Thermofisher) and cocultured with BM-MDSCs and CD3/28 dynabeads (Thermofisher) at 1:1:1 ratio in 200 µl complete RPMI media for 72 hrs. Cells were harvested, stained for CD4 and CD8, and analyzed for proliferation in flow cytometer (BD Fortessa).

### Bone marrow derived macrophage differentiation

*In vitro* macrophages were derived by culturing bone marrow cells in complete RPMI supplemented with recombinant mouse M-CSF (20 ng/ml; Biolegend) for 6 days with media replaced at day 4. At day 6 adherent cells were harvested with TrpLE (Thermofisher) and replated in 6 well plate (1x10^6^ cells per well) in 2 ml complete RPMI media without M-CSF. After overnight culture, the cells were either treated with WP1066 (4 µM) or DMSO for 24 hrs. Cells were then harvested and stained for macrophage markers including CD11b, F4/80, MHC-II, and CD80 and analyzed by flow cytometer.

### Flow cytometry

Lungs draining cervical lymph nodes (LDLN) and lungs were harvested from mice at day 16 post tumor cell injection for analysis. Single cells suspensions from the LDLN were prepared by mashing the tissue through 70 µm cell strainer. Single cells from lungs were prepared by enzymatic digestion of finely chopped lung tissues in complete RPMI supplemented with collagenase-IV (Worthington) and DNAse (Sigma) at 37°C for 30mins. Digested tissues were then mashed through 70 µm cell strainer to obtain single cell suspensions. Red blood cells were lysed using RBC lysis buffer (Biolegend). Single cells from lymph nodes and lungs were washed with PBS and stained with Fixable Zombie violet dye for 10 mins at 4 C in the dark. Cells were then stained with Fc block (CD16/32 antibody) and stained for surface markers with a combination of antibodies (**Supplementary Table 1**) diluted in FACS buffer (PBS supplemented with 1% BSA and 0.05% NaN3) with Brilliant Violet stain buffer for 30 mins at 4 C in the dark. After incubation, cells were washed in FACS buffer and fixed using fixation buffer (Thermofisher). For intracellular/intranuclear staining, fixed cells were washed with permeabilization buffer followed by staining with antibody in the buffer for 30 mins at 4 C. Cells were acquired using BD Fortessa and analyzed using FlowJo v. 10 software.

### Cytokine analysis

Single cells from lungs and LDLNs (2x10^6^) were activated with phorbol 12-myristate 13-acetate (PMA, 50 ng/ml) and Ionomycin (500 ng/ml) for 4 hrs in presence of Brefeldin (Biolegend) and Monensin (Biolegend). Cells were harvested after 4 hrs and stained as described before. Cells were stained for surface markers followed by intracellular for IFN-γ and TNF-α.

### Statistical analysis

All statistical analysis was performed in Graph Pad Prism v. 9. Data are represented as mean ± standard error of the mean (SEM). For comparison between two groups, an unpaired two-tailed student’s t-test or Mann-Whitney test was used as indicated in figure legends. One way ANOVA followed by Tukey’s multiple comparison test was performed for comparing more than two groups. Survival analysis was performed using the log-rank (Mantel-Cox) test. p-value of <0.05 was considered statistically significant.

### Data availability

Data generated in the study are included in the manuscript and the supplementary information file. The raw data are available upon request from the corresponding author.

## Results

### Effect of WP1066 on tumor cell proliferation, apoptosis, and phagocytosis by macrophages

First, we evaluated the status of activated STAT3 in OS cell lines. We performed western blot analysis on cellular lysates of multiple metastatic and non-metastatic mouse and human OS cell lines. In line with previous reported observations, phosphoSTAT3 (pSTAT3^Y705^) was detected at different intensities in all tested cell lines (**Supplementary Figures 1A, B**). We next investigated if WP1066 treatment inhibits STAT3 activation in OS cell lines. Mouse (K7M3 and DLM8) and human OS cells (OS17 and LM7) were treated with different concentrations of WP1066. WP1066 treatment for 24 hrs reduced the level of pSTAT3^Y705^ in a dose dependent manner as compared to DMSO-treated cells (**Supplementary Figures 1C, D**). This suggests that pSTAT3^Y705^ is constitutively expressed in OS cells and that WP1066 inhibits activated STAT3.

**Figure 1:**
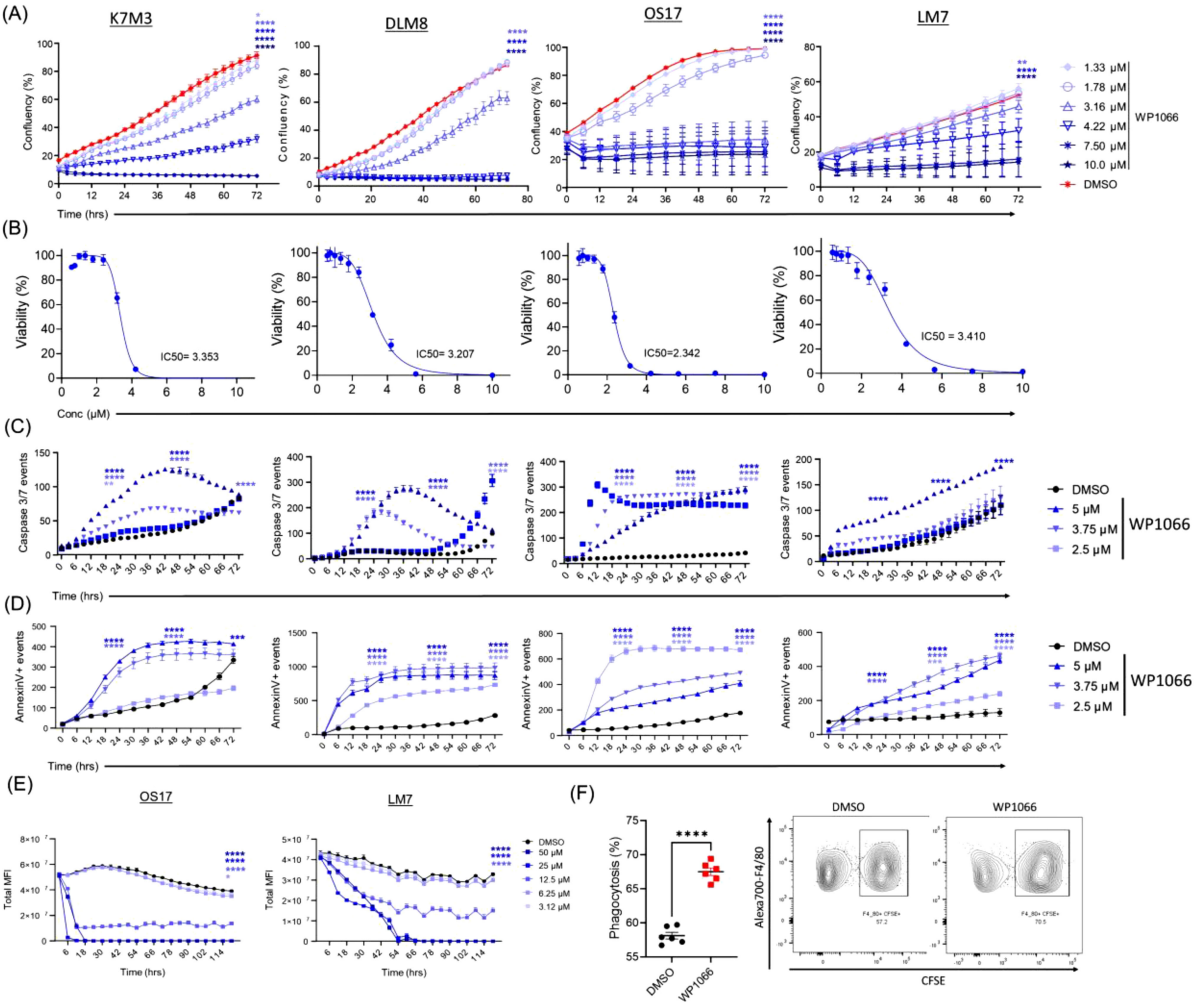
WP1066 inhibited cell proliferation, induced apoptosis, and increased phagocytosis of OS cells. **(A)** Cell confluency assay. Mouse and human OS cells were incubated with the indicated concentration of WP1066 and assessed for confluency using the Incucyte S3 for 72 hrs. **(B)** Resazurin assay. After 72 hrs, cells from **(A)** were incubated with Resazurin for 2 hrs. Fluorescence of resorufin was measured to calculate the IC50 values. **(C)** Kinetics of Caspase-3/7 activation following WP1066 treatment. OS cells were cultured with various concentrations of WP1066 and caspase-3/7 green dye. Green fluorescence (indicating activated caspase-3/7) was measured with the Incucyte S3 for 72 hrs. **(D)** Annexin V kinetics assay. OS cells were cultured with WP1066 and Annexin V-green reagent for 72 hrs. Green fluorescence (indicating expression of Annexin V) was measured for 72 hrs. **(E)** Effect of WP1066 on OS tumor spheroids. OS17-GFP and LM7-GFP generated tumor spheroids were treated with different concentrations of WP1066. Green fluorescence (indicating the viability of the spheroids) was measured at 3 hr intervals for 72 hrs. **(F)** Phagocytosis of K7M3 OS cells by RAW264.7 cells. CFSE-labelled DMSO and WP1066 (2.5 μM) treated K7M3 cells were cocultured with RAW264.7 mouse macrophage cells for 4 hrs and analyzed by flow cytometry for CFSE^+^F4/80^+^ cells. Two-way ANOVA followed by Tukey’s multiple comparison test was used for statistical analysis in **(A, C, D, E)**. Statistical significance compared to DMSO control is shown at 72 hrs time point for **(A, E)**, and at 24, 48 and 72 hrs for **(C, D)**. Mann-Whitney test was used in **(F)**. Data points are shown as mean ± SEM, representative of two independent experiment. *p<0.05, **p<0.01, ***p<0.001, ****p<0.0001.

Given the critical role of STAT3 in cell proliferation in multiple tumor models, we investigated the effect of inhibiting STAT3 activation by WP1066 in OS cells. To determine if WP1066 had a cytotoxic effect, mouse (K7, K7M3, Dunn, DLM8) and human (OS17, LM7, MG63, MG63.2, 143B, HOS) OS cells were incubated with different concentrations of WP1066 for 72 hrs and analyzed for cell viability by resazurin assay (**Figure 1A**; **Supplementary Figure S1E**). WP1066 inhibited proliferation of both mouse and human OS cells in a concentration dependent manner (**Figures 1A, B**). The IC_50_ against mouse OS cells ranged from 2.79 µM to 6.01 µM with K7M3 being more sensitive than K7 (**Figure 1B**; **Supplementary Figure S1E**). The IC50 for human OS cells ranged from 2.3 µM to 3.5 µM with OS17 being more sensitive than LM7 (**Figure 1B**; **Supplementary Figure S1E**). Moreover, we assessed if WP1066 induced tumor cell apoptosis using a fluorescence-based assay to assess the kinetics of activation of caspase-3/7. WP1066 treatment activated caspase-3/7 across all tested cell lines. The higher dose of WP1066 (5 µM) induced swift activation of caspase-3/7, whereas a gradual increase over time was observed when cells were treated with the lower dose of WP1066 (2.5 µM) compared to DMSO (**Figure 1C**). As another measure for apoptosis, we also assessed the level of PS by Annexin V. Similar kinetics for the expression of PS (Annexin V^+^) was observed following treatment with WP1066, supporting the conclusion that WP1066 induced apoptosis (**Figure 1D**).

We also investigated WP1066-induced toxicity using tumor spheroids. Tumor spheroids were generated from OS17-GFP and LM7-GFP cells and were treated with different concentrations of WP1066. Without WP1066 treatment, OS17 and LM7 derived tumor spheroids remained stable and maintained strong GFP-mean fluorescence intensity (MFI) throughout the duration of the experiment, indicating viability. By contrast, the cytotoxicity of WP1066 against tumor spheroids was demonstrated by a significant drop in GFP-MFI in a concentration dependent manner (**Figure 1E**).

WP1066-induced apoptosis may upregulate the expression of “eat me signals” which have been shown to enhance phagocytosis (47). To determine if WP1066 enhanced tumor cell phagocytosis, K7M3 cells were treated with WP1066 (2.5 µM) for 24 hrs. CFSE-labelled WP1066- or DMSO-treated K7M3 cells were co-cultured with RAW264.7 mouse macrophages for 4 hrs. Flow cytometric analysis revealed that there was a significant increase in the phagocytosis of WP1066-treated K7M3 cells compared to those treated with DMSO (**Figure 1F**). This data supports that WP1066 treatment enhanced the ability of macrophages to phagocytose tumor cells. Taken together, these data suggest that WP1066 suppresses cell proliferation, induces apoptosis, and enhances phagocytosis.

### WP1066 activates macrophages and inhibits the immunosuppressive activity of MDSCs

**Figure 2:**
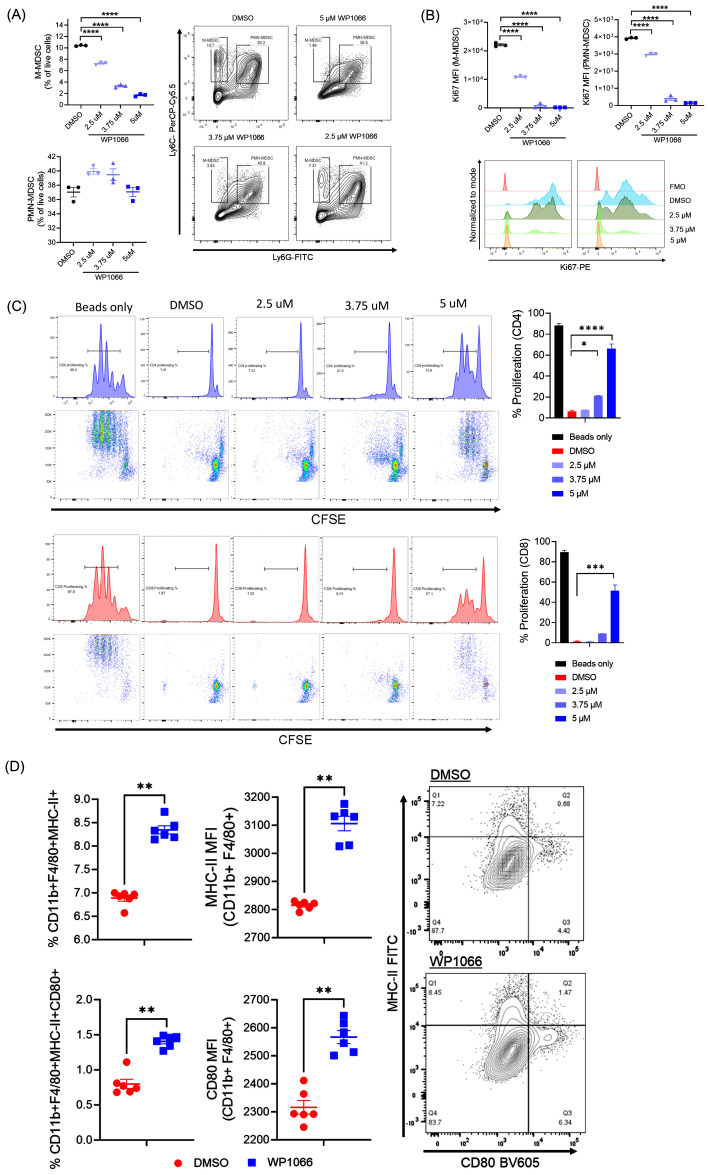
Effect of WP1066 on BM-MDSCs and BMDM. **(A, B)** WP1066 decreased the viability and inhibited the proliferation of BM-MDSCs. *In vitro* bone marrow-generated MDSCs were cultured with the indicated concentration of WP1066 for 24 hrs. Flow cytometric analysis was done to quantify viability **(A)** and Ki67 expression **(B)** of M-MDSCs (CD11b^+^Ly6C^+^Ly6G^-^) and PMN-MDSCs (CD11b^+^Ly6C^+^Ly6G^+^) cells. **(C)** Suppression of T-cell proliferation by WP1066-treated BM-MDSCs. BM-MDSCs were treated with DMSO or WP1066 for 24 hrs and co-cultured with CFSE labelled T-cells and CD3/CD28 dyna beads. After 72 hrs of co-culture, cells were analyzed for proliferation by flow cytometry. **(D)** Activation of BMDM by WP1066. Macrophages derived from bone marrow were incubated with DMSO or WP1066 for 24 hrs. Cells were then harvested and analyzed by flow cytometry for activation by expression of MHC-II and CD80. Statistical significance was determined using one-way ANOVA followed by Tukey’s multiple comparisons test in **(A-C)**, and the Mann-Whitney test in **(D)**. Data points shown as mean ± SEM. *p<0.05, **p<0.01, ***p<0.001, ****p<0.0001.

We next sought to investigate the effect of WP1066 on MDSCs and macrophages generated from bone marrow cells. First, we assessed the effect of WP1066 on viability and proliferation of MDSCs. *In vitro* generated BM-MDSCs were treated for 24 hrs with WP1066 and then analyzed by flow cytometry for the markers CD11b, Ly6C, and Ly6G to differentiate between monocytic MDSCs (M-MDSCs, CD11b^+^Ly6C^hi^Ly6G^-^) and polymorphonuclear MDSCs (PMN-MDSCs, CD11b+Ly6C+Ly6G+) (45). WP1066 treatment significantly reduced the frequency of live M-MDSCs as compared with the DMSO treated control (**Figure 2A**). However, there was no statistical difference in percentage of live PMN-MDSCs between the treated and control groups. When analyzed for the expression of Ki67, a proliferation marker, WP1066 significantly reduced the mean fluorescence intensity (MFI) of Ki67 in both M-MDSCs and PMN-MDSCs (**Figure 2B**). These data indicate that M-MDSCs are more susceptible to the *cytotoxic* effect of WP1066 as compared to PMN-MDSCs. However, WP1066 suppressed the proliferation of both M-MDSCs and PMN-MDSCs.

Next, we evaluated whether the immunosuppressive capacity of MDSCs was affected by WP1066. BM-MDSCs were treated with WP1066 for 24 hrs and then evaluated for T cell suppression capacity by co-culture assay. DMSO treated BM-MDSCs suppressed the proliferation of CFSE-labelled T cells. CFSE^low^ daughter generation was not observed as compared with T cells activated with dynabeads alone. In contrast, CD4 and CD8 T cell proliferation was increased when co-cultured with BM-MDSCs treated with WP1066 in a dose dependent manner (**Figure 2C**). These observations are in line with other studies where inhibition of STAT3 has been shown to induce apoptosis and attenuate the suppressive capacity of MDSCs (48, 49). Taken together, these data suggest that WP1066 significantly affects the viability, proliferation, and suppressive capacity of BM-MDSCs.

Given the significant role of STAT proteins in plasticity and differentiation of macrophages (50), we next determined the *in vitro* effect of WP1066 on BMDM. WP1066 treatment for 24 hrs significantly increased the frequency of macrophages expressing MHC-II (CD11b^+^F4/80^+^MHC-II^+^) and co-expressing MHC-II and CD80, markers associated with M1-like or proinflammatory phenotype (**Figure 2D**). In addition, WP1066 increased the MFI of CD80 and MHC-II in macrophages as compared with DMSO control (**Figure 2D**). The increase in MHC-II and co-stimulatory molecule CD80 on macrophages following WP1066 treatment suggests that STAT3 inhibition by WP1066 activates macrophages and enhances antigen presentation. Taken together, the described effects on MDSCs and macrophages indicates a potential positive immunomodulatory effect by WP1066 on myeloid cells through suppression of MDSCs and activation of anti-tumor macrophage function.

### Effect of WP1066 on survival of mice with OS lung metastasis

**Figure 3:**
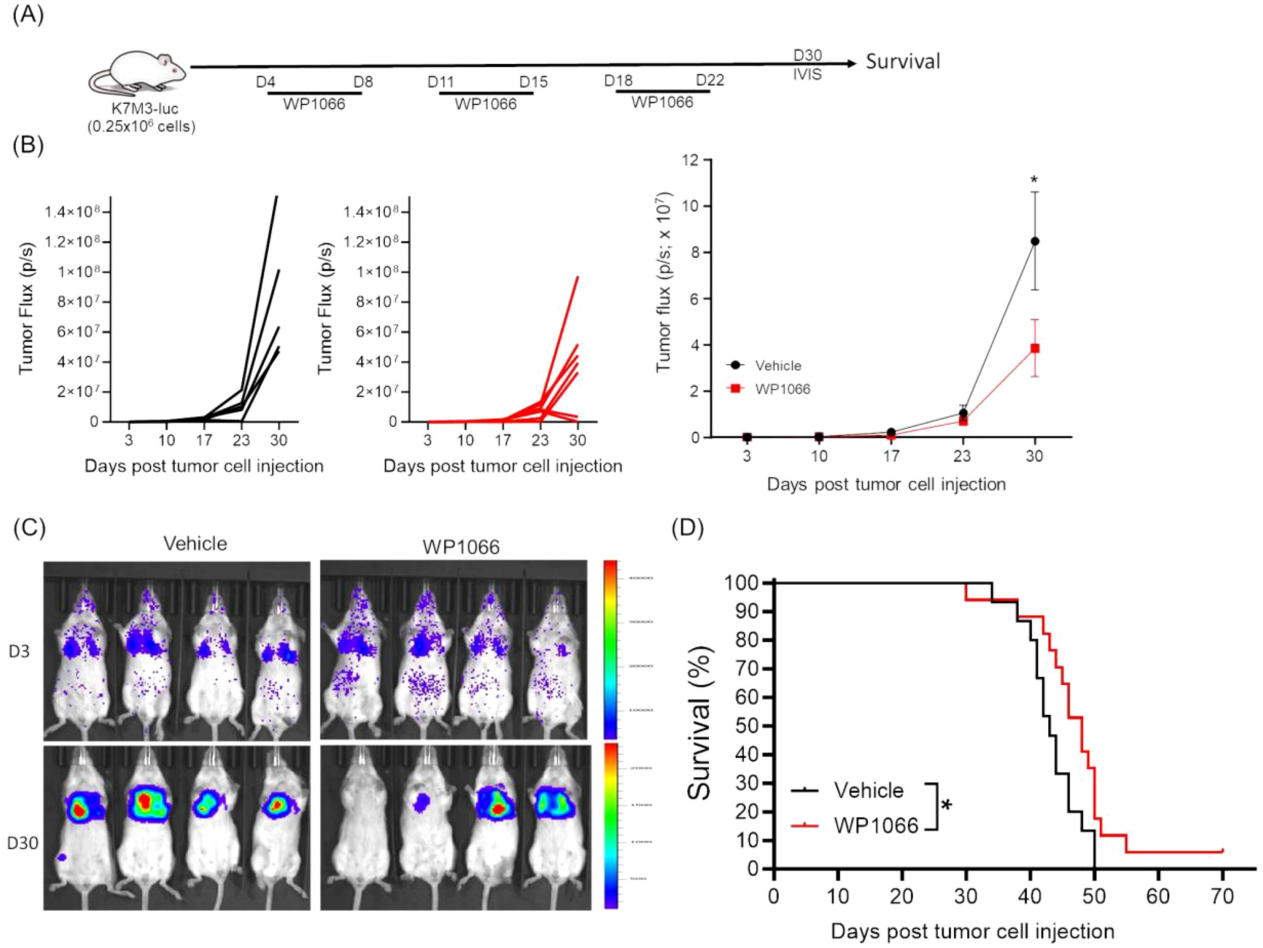
Effect of WP1066 on OS lung metastasis. **(A)** Experimental schema. **(B)** Individual and average tumor growth over time. **(C)** IVIS imaging at day 3 and 30 after tumor cell injection. **(D)** Overall survival curves of mice treated with vehicle (n=15) or WP1066 (n=17). Survival curve comparison was done with log-rank test. Data points shown as mean ± SEM. *p<0.05.

The syngeneic K7M3 experimental metastasis model was used to determine the therapeutic effect of WP1066 on established lung metastases. K7M3-luc cells were injected intravenously (i.v) and lung metastases were validated by IVIS imaging before treatment on day 3 post tumor cell injection. Mice were treated with WP1066 (o.g) or vehicle control 24 hrs after imaging (**Figure 3A**). WP1066 treatment significantly reduced the tumor growth (**Figures 3B, C**) and improved the survival. The median survival time (MST) for WP1066-treated cohort was 48 days as compared to 42 days for the control group (p=0.037). Interestingly, 1 out of 17 mice treated with WP1066 survived more than 70 days, whereas all control mice died by day 50. This suggests the potential therapeutic activity of WP1066 against OS-lung metastases.

### Effect of combining anti-CD47 with WP1066 on survival

**Figure 4:**
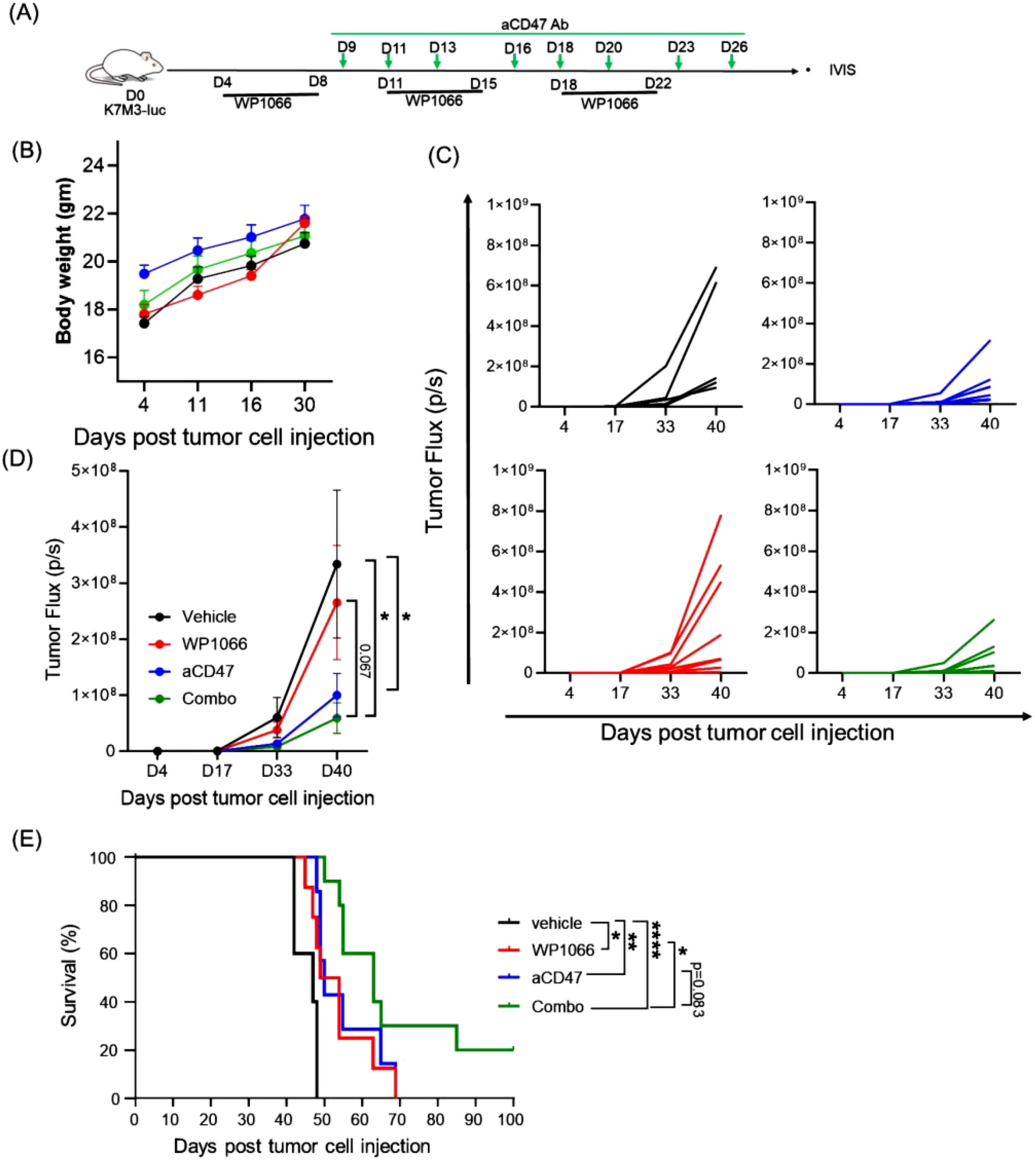
Effect of combining WP1066 with anti-CD47 against OS lung metastasis. **(A)** Experimental schema. **(B)** Body weight **(C)** Tumor growth of individual mice as quantified by IVIS imaging. **(D)** Average tumor growth for each group. **(E)** Long-term survival curves (n=5-10). Statistical significance calculated for tumor growth at day 40 by Mann Whitney test in **(D)** and survival curves were compared using log-rank test in **(E)**. Data points shown as mean ± SEM. *p<0.05, **p<0.01, ****p<0.0001.

WP1066 treatment resulted in only a modest 6-day increase in MST, highlighting limited activity. We reasoned that combining WP1066 with immunotherapy could enhance therapeutic efficacy. The CD47-SIRPα axis, also referred to as an innate immune checkpoint, regulates the activation of innate immune cells (24). Neutralizing CD47-SIRPα axis with anti-CD47 antibody enhances tumor phagocytosis by macrophages, and activation of Natural Killer cells (NK), Dendritic cells (DCs), neutrophils and macrophages (24–26). In line with previous reports, flow cytometric and immunoblot analysis revealed that both human and mouse OS cells constitutively express CD47 (**Supplementary Figure S2**), suggesting a potential mechanism of immune evasion (51). These findings prompted us to investigate whether combining WP1066 with anti-CD47 antibody would improve the anti-tumor responses. Tumor bearing mice were treated with WP1066 alone, anti-CD47 alone or WP1066 + anti-CD47 (**Figure 4A**). No significant change in body weight was observed in any of the groups (**Figure 4B**). Combination therapy significantly inhibited tumor growth (p=0.028) and prolonged survival (p<0.0001) compared to the vehicle treated control cohort (**Figures 4C–E**). The MST for mice that received combination therapy was 63 days compared to 47 days for the control group. Monotherapy with anti-CD47 also resulted in a significant decrease in tumor growth (p=0.048, **Figure 4D**) and an increase in MST (p<0.001, **Figure 4E**) compared to control. While there was a significant increase in MST in mice treated with combination therapy compared to those treated with WP1066 alone (p=0.027), the improvement in MST between mice treated with combination therapy versus anti-CD47 alone did not reach statistical significance (MST: 63 days vs 50 days, p =0.083). However, using the hazard ratio (log-rank test) to compare the anti-CD47-treated group with the combinatorial therapy group indicated that the mice treated with the monotherapy had more than twice the risk of death compared to those receiving combination therapy (Hazard ratio: 2.237, 95% CI: 0.7257 to 6.894). This represents a ~55% reduction in risk with the combination therapy, which is biologically meaningful. These data suggest that combination therapy can improve the anti-tumor response of WP1066 against established lung metastases.

### Combination treatment increases infiltration of activated CD8^+^ T, NK, and macrophages

**Figure 5:**
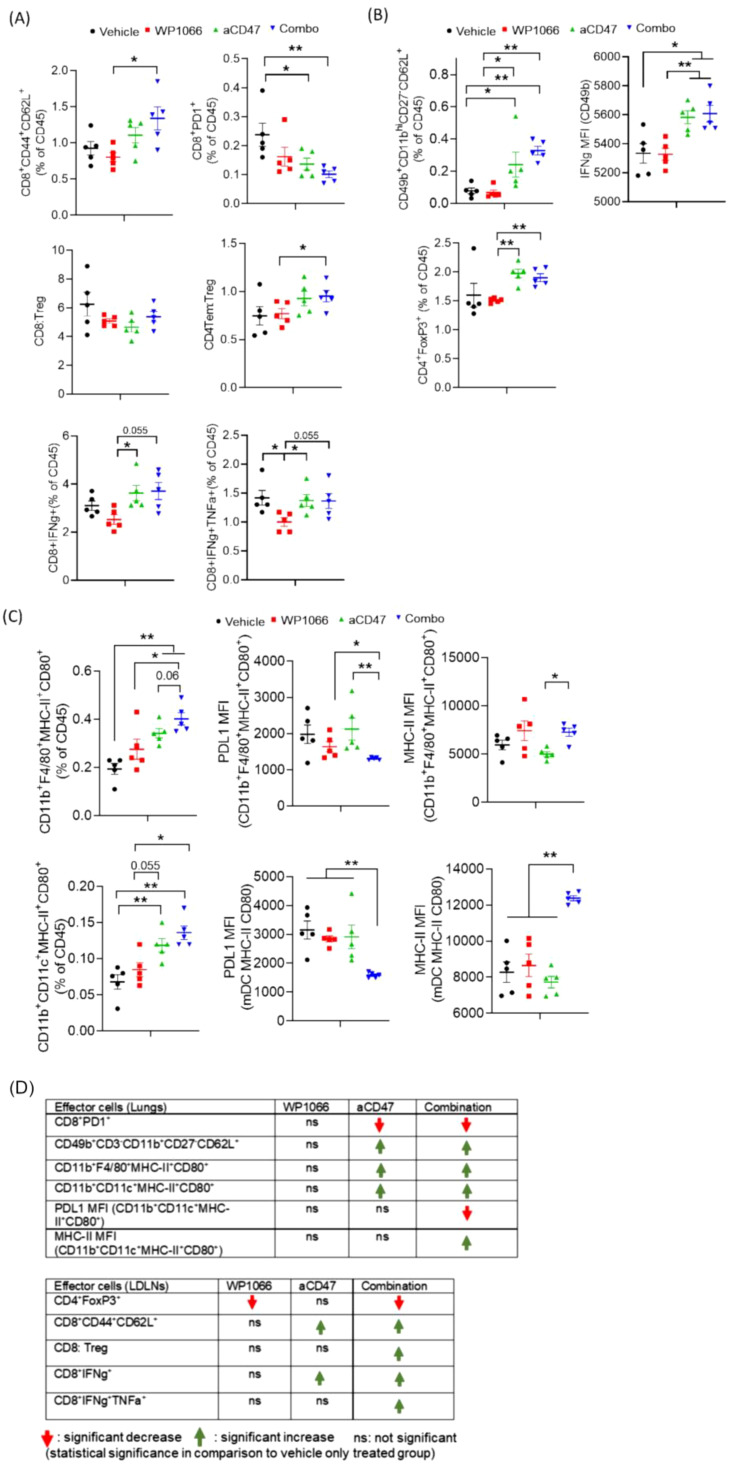
Effect of WP1066 plus anti-CD47 on immune cell infiltrates in lungs. **(A)** Flow cytometric analysis on single cells from lungs for **(A)** central memory CD8 T cells (CD8^+^CD44^+^CD62L^+^), CD8^+^PD1^+^, IFN-g and TNF-a secreting CD8^+^ T cells, **(B)** NK cells (CD49b^+^CD11b^hi^CD27^-^CD62L^+^), FoxP3 expressing Tregs (CD4^+^FoxP3^+^), **(C)** Activated CD11b^+^F4/80^+^ macrophages and CD11b^+^CD11c^+^ dendritic cells co-expressing MHC-II and CD80. MFI of PDL1 and MHC-II in macrophages and dendritic cells in lungs. **(D)** Summary of changes in indicated effector cells in lungs and LDLNs. Statistical significance calculated using Mann-Whitney Test. Data points shown as mean ± SEM. *p<0.05, **p<0.01.

As the therapeutic efficacy of immunotherapy has been shown to be linked to immune cell infiltration into the tumor and tumor draining lymph nodes, we performed flow cytometric analysis of lungs and LDLNs at day 16 post tumor cell injection (**Figure 5A**; **Supplementary Figure S3**). Analysis of lungs revealed that the combination treatment increased central memory CD8^+^ T cells (CD8Tcm; CD8^+^CD44^+^CD62L^+^) (**Figure 5A**). In addition, combination treatment significantly reduced PD1 expressing CD8 T cells (CD8^+^PD1^+^), a marker of dysfunction when compared with vehicle treated control cohort (**Figure 5A**). While combination treatment also increased FoxP3^+^ expressing regulatory T cells (CD4^+^FoxP3^+^; Tregs) the CD4 Tem (CD4^+^CD44^+^CD62L^-^): Treg ratio, was increased suggesting increased number of effector memory CD4^+^T cells to Tregs. Furthermore, analysis of cytokine secreting cytotoxic CD8^+^ T cells revealed that combination treatment increased IFN-γ secreting CD8^+^ T cells, a marker of activated T-cells (**Figure 5A**). Analysis of cytotoxic NK cells in lungs suggested that combination therapy increased the level of differentiated mature NK cells (CD49b^+^CD11b^hi^CD27^-^CD62L^+^). Furthermore, IFN- γ expression in NK cells was significantly increased in mice that received both the treatments (**Figure 5B**). Interestingly, we observed a similar trend in animals treated only with anti-CD47 antibody (**Figures 5A, B**).

Analysis of antigen presenting cells (APCs) infiltrating the lungs revealed that combination treatment significantly increased the frequency of M1-like or proinflammatory activated macrophages (CD11b^+^F4/80^+^MHC-II^+^CD80^+^) versus control cohort (**Figure 5C**). Combination therapy also increased activated myeloid derived monocytic DCs co-expressing MHC-II and CD80 (CD11b^+^CD11c^+^MHC-II^+^CD80^+^). Furthermore, APCs infiltrating the lungs of animals in the combination cohort had significantly reduced expression of PDL1 (associated with immune suppression) and increased expression of MHC-II (**Figure 5C**).

These observations were consistent with the immune cell profile in the LDLNs. LDLN from animals treated with both agents had an increased CD8: Treg ratio with significantly increased CD8 Tcm and reduced CD4^+^ Tregs and reduced dysfunctional CD8^+^PD1^+^ (**Supplementary Figure S3**). Mice treated with combination therapy also had a significant increase in cytotoxic CD8 T cells co-secreting IFN-γ and TNF-α (**Supplementary Figure S3A**).

Taken together, as summarized in **Figure 5D**, these data suggest that the therapeutic efficacy of combination therapy was associated with an augmented pro-inflammatory anti-tumor immune response mediated by increased infiltration of activated immune cells into the lungs and LDLNs, including IFN-γ secreting cytotoxic CD8 T cells, NK cells and activated macrophages and monocytic DCs. These pro-inflammatory effects were paralleled with decreased immune suppression as observed by a decrease in Tregs and dysfunctional PD1 expressing CD8 T cells in combination treatment cohort.

### Efficacy of combination therapy on established OS lung metastases using an orthotopic OS mouse model

**Figure 6:**
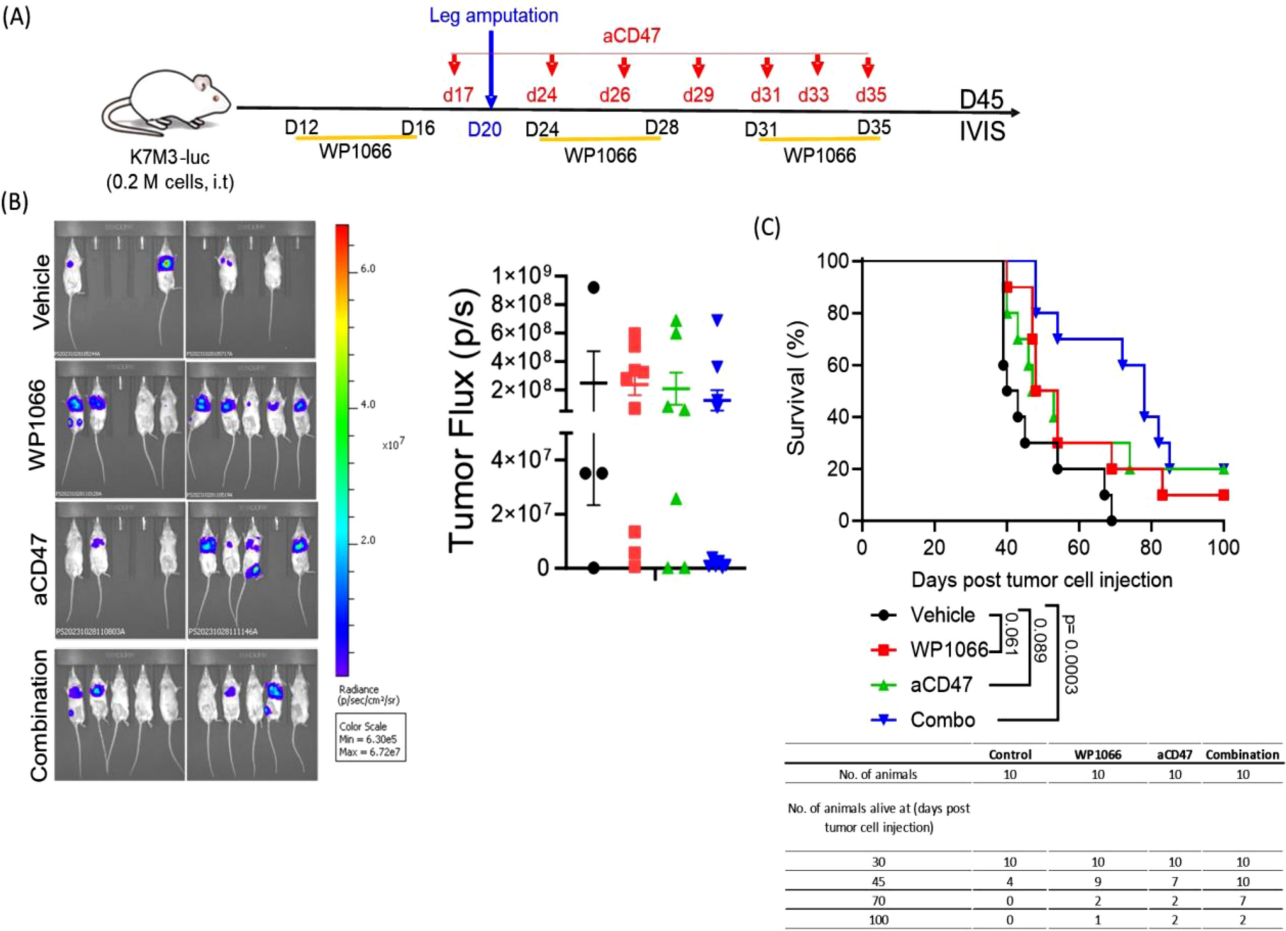
Therapeutic efficacy of combination treatment using the orthotopic lung metastasis model. **(A)** Experimental schema. **(B)** Representative bioluminescence images of lung metastases at day 45 and tumor flux. **(C)** Survival curve (n=10). Statistical significance for survival was determined using the log-rank test. Data points shown as mean ± SEM.

Next, we evaluated the therapeutic effect of combination therapy using our orthotopic intra-tibial OS model in which lung metastasis form *spontaneously* from a primary tumor in the leg (38). This model better reflects how lung metastases develop in patients. For this purpose, K7M3-luc cells were injected into the right tibia of BALB/c mice. Mice were treated with the first cycle of therapy using WP1066, CD47 or combination therapy as shown in **Figure 6A**. Amputation of the leg-bearing primary tumor was performed 3 weeks after tumor cell injection to prevent further metastatic spread. This was followed by the treatment strategy shown in the schema (**Figure 6A**). Although the lung tumor burden, as represented by tumor flux, at day 45 post tumor cell injection was not statistically significant among the groups (**Figure 6B**), there was a significant difference in the survival time. In control group, 60% of mice died by day 45 and 100% had to be euthanized by day 70. By contrast the death rates were 10% and 30% in mice treated with WP1066 and anti-CD47 respectively. There were no deaths in the combination therapy group at 45 days. Survival at day 70 was 0%, 20%, 20% and 70% in control, WP1066, anti-CD47, and combination therapy treated mice respectively (**Figure 6C**). The MST of the mice treated with combination therapy was 78 days compared to 51 and 50 days for mice treated with WP1066 and anti-CD47 respectively. These data suggest that combined treatment with WP1066 and anti-CD47 is also more effective in prolonging survival in an orthotopic OS model.

## Discussion

The overall survival of recurrent and relapse osteosarcoma patients has not improved and remains a challenge. Addition of drugs to standard chemotherapy regimens has failed to improve the 60-65% survival rate and there has been no improvement in survival outcome of patients with lung metastases (3, 52, 53). Moreover, immunotherapy targeting T-cells showed disappointing response rates due to the immunosuppressive microenvironment (2, 54, 55). In this study, we showed that OS cells constitutively express phosphoSTAT3 and that the orally bioavailable STAT3 inhibitor WP1066 reduced STAT3 activation. WP1066 treatment induced activation of caspase-3/7 and PS expression on the outer leaflet of the cell membrane indicating its cytotoxic effect against both mouse and human OS cells. This cytotoxic effect was also observed on tumor spheroids. In addition to direct cytotoxic effect on tumor cells, WP1066 also inhibited the proliferative capacity of both M-MDSCs and PMN-MDSCs and induced apoptosis of M-MDSCs as quantified by flow cytometry. Finally, WP1066 decreased MDSC-immunosuppressive function against T cells. These changes can have a significant impact on decreasing the immunosuppressive TME, thereby improving response to T-cell directed therapy.

Activation of macrophage-mediated anti-tumor activity has been shown to correlate with improved survival in OS patients (18–20) and there is also a correlation between anti-tumor M1/proinflammatory macrophage content in OS lung metastases and improved survival (56). Phenotypic switching of intra-tumor macrophage from M2 to M1 has been shown to inhibit OS tumor growth in a mouse model (57). Therefore, inducing an increase in anti-tumor macrophage function is anticipated to be beneficial in improving the response to immunotherapy. WP1066 not only inhibited MDSCs but increased macrophage-mediated phagocytosis of tumor cells. Such changes are anticipated to contribute to an *immune-supportive* TME.

When evaluated *in vivo*, WP1066 monotherapy prolonged the survival of mice with established lung metastases. This therapeutic response was significantly improved when anti-CD47 antibody was added to the treatment. These results demonstrate that combining a STAT3 inhibitor that decreases MDSCs (48) with an anti-CD47 antibody that enhances macrophage function (28, 58) may produce a synergistic response resulting in a better therapeutic outcome.

Studies have confirmed that the dysregulated activation of STAT3 in multiple tumors plays a critical role in different aspects of oncogenesis and tumor-immune evasion (7, 15). STAT3 is consistently activated in multiple human tumors including osteosarcoma (4). Tumor cells upregulate activated STAT3 to enhance cell cycle progression, angiogenesis, and prevent apoptosis. STAT3 regulates epithelial mesenchymal transition and plays an important role in metastasis as well as chemotherapy sensitivity (12, 59, 60). WP1066 is a commercially available STAT3 inhibitor derived from caffeic acid (29, 30). WP1066 has been evaluated for its therapeutic efficacy against a wide range of tumors including gliomas, leukemia, melanoma, breast cancer, renal cancer and others (29, 43, 61–63). In line with previous reports, we show that STAT3 is constitutively activated in multiple OS cell lines and that WP1066 inhibits the activation. Our data show that WP1066 suppressed OS cell proliferation, induced apoptosis, and suppressed the growth of tumor spheroids. Besides tumor intrinsic effects, activated STAT3 also has been shown to inhibit the activity of T-cell directed immunotherapy by supporting T-regs, immunosuppressive macrophages and MDSCs in the TME. WP1066 has been reported to inhibit regulatory T cells (43). Our data suggest that targeting STAT3 will modulate the immunosuppressive TME and enhance anti-tumor immunity. We show here for the first time that in addition to its effect on T-regs, WP1066 was cytotoxic to M-MDSCs, inhibited their proliferation and attenuated the MDSC-suppressive capacity while increasing MHC-II, and CD80, markers of activated macrophages. These changes were accompanied by an increase in median and overall survival time.

The CD47-SIRPα pathway is an innate immune checkpoint that regulates activation of innate immunity. Tumor cells overexpress CD47 to inhibit macrophage phagocytosis and immune activation (25, 64, 65). Therefore, blocking the CD47-SIRPα axis potentially activates adaptive immunity by enhancing the cross-presentation of antigens (28). We hypothesized that combining WP1066 (which inhibits MDSCs and activates macrophages) with anti-CD47 antibody (which activates innate immunity) will further enhance anti-tumor immunity and therapeutic activity against OS lung metastases. Indeed, the addition of anti-CD47 to WP1066 significantly improved the survival as compared to monotherapy. The survival benefit from anti-CD47 monotherapy was comparable to WP1066 monotherapy and is in line with previously published investigations in OS (51, 58, 66). Importantly, combination therapy was significantly better than monotherapy. In addition to improving survival, the dual treatment altered the immune landscape by increasing the activated cytotoxic CD8^+^ T cells in lungs as well as the LDLNs. This was associated with a significant drop in CD8^+^PD1^+^ T cells associated with dysfunctional CD8^+^ T cells. Combination treatment increased the CD8:Treg and CD4Tem: Treg ratio in LDLN and lung respectively, indicating fewer Tregs in comparison to the number of CD4 and CD8 T cells. In addition, mice treated with combination therapy had a significant increase in activated macrophages and monocytic DCs in the lungs, consistent with inflammatory activated APCs with increased capacity for antigen presentation. In addition, these APCs had a significantly lower level of PDL1, an immunosuppressive ligand known to maintain a suppressive TME. Collectively, these data suggest that combining a STAT3 inhibitor with CD47-SIRPα blockade will positively modulate the TME and enhance the therapeutic response.

The combination therapy resulted in a low rate of long term (>100 days) survivals, potentially limited by the brief treatment regimen. The effect of extending the duration of treatment on survival rates is unknown and needs to be investigated. Future research should prioritize identifying the immune effector cells and the mechanisms driving the additive effect. Furthermore, it will also be important to investigate the impact of STAT3 inhibition on other cells in TME such as stromal cells, fibroblasts, and endothelial cells.

In summary, the data presented here demonstrated that STAT3 and CD47 are two potential therapeutic targets for the treatment of OS lung metastases. While inhibiting either pathway alone resulted in therapeutic benefits, combined treatment resulted in the best improvement in survival (MST and long-term survival). Furthermore, our data support the hypothesis that the improved therapeutic benefits in mice receiving combination therapy was secondary to the alteration of the TME with increased infiltration of cytotoxic anti-tumor immune cells including T cells, NK cells, and activated macrophages and DCs. To our knowledge, we are the first to evaluate this combination therapy in the context of OS lung metastases and to show that the positive changes in the immune landscape of the TME and LDLNs with regard to immune cell content, corresponds to the anti-tumor therapeutic activity. These studies provide pre-clinical justification for further exploring this combination for the treatment of patients with metastatic OS in the lungs.

Both WP1066 and anti-CD47 antibody therapies have demonstrated favorable safety profiles in preclinical and early-phase clinical studies. WP1066 was recently evaluated in Phase I clinical trial in pediatric patients with malignant brain tumors (NCT04334863); although the study has been completed, results have not yet been published (67). In adult patients, the most common adverse event in adult patients treated with WP1066 was grade 1 nausea and diarrhea in 50% of patients with no significant hematological toxicity (29). Notably, WP1066 has received FDA Rare Pediatric Disease Designation for three pediatric brain cancers, highlighting its potential use against pediatric cancers (68). Anti-CD47 therapy has been evaluated against hematologic and solid tumors in adult patients. The anti-CD47 therapy is associated with transient anemia, fatigue and no severe non-hematologic toxicities (69). The therapy has not yet been tested in clinical trials for pediatric patients. However, because of recent safety concerns FDA has placed hold on clinical trials evaluating anti-CD47 therapy (70). In our pre-clinical mouse model, the combination was well tolerated with no significant weight loss or observable effects. Although pediatric clinical data for this combination are limited, the existing evidence supports its translational potential in pediatric OS with appropriate safety monitoring.

## Data Availability

The original contributions presented in the study are included in the article/**Supplementary Material**. Further inquiries can be directed to the corresponding author.

## References

[1] Meltzer PS Helman LJ. New horizons in the treatment of osteosarcoma. New Engl J Med. (2021) 385:2066–76. doi: 10.1056/NEJMra2103423 34818481

[2] Beird HC Bielack SS Flanagan AM Gill J Heymann D Janeway KA. Osteosarcoma. Nat Rev Dis Primers. (2022) 8:77. doi: 10.1038/s41572-022-00409-y 36481668

[3] Kleinerman E. Maximum benefit of chemotherapy for osteosarcoma achieved—What are the next steps? Lancet Oncol. (2016) 17:1340–2. doi: 10.1016/S1470-2045(16)30270-4 27569441

[4] Yu H Lee H Herrmann A Buettner R Jove R. Revisiting STAT3 signalling in cancer: new and unexpected biological functions. Nat Rev Cancer. (2014) 14:736–46. doi: 10.1038/nrc3818 25342631

[5] Bromberg JF Wrzeszczynska MH Devgan G Zhao Y Pestell RG Albanese C. Stat3 as an oncogene. Cell. (1999) 98:295–303. doi: 10.1016/s0092-8674(00)81959-5 10458605

[6] Johnson DE O’Keefe RA Grandis JR. Targeting the IL-6/JAK/STAT3 signalling axis in cancer. Nat Rev Clin Oncol. (2018) 15:234. doi: 10.1038/nrclinonc.2018.8 29405201 PMC5858971

[7] Huynh J Chand A Gough D Ernst M. Therapeutically exploiting STAT3 activity in cancer — Using tissue repair as a road map. Nat Rev Cancer. (2019) 19:82–96. doi: 10.1038/s41568-018-0090-8 30578415

[8] Liu Y Liao S Bennett S Tang H Song D Wood D. STAT3 and its targeting inhibitors in osteosarcoma. Cell Prolif. (2020) 54:e12974. doi: 10.1111/cpr.12974 33382511 PMC7848963

[9] Fossey SL Liao AT McCleese JK Bear MD Lin J Li P-K. Characterization of STAT3 activation and expression in canine and human osteosarcoma. BMC Cancer. (2009) 9:81. doi: 10.1186/1471-2407-9-81 19284568 PMC2666757

[10] Zhang T Li J Yin F Lin B Wang Z Xu J. Toosendanin demonstrates promising antitumor efficacy in osteosarcoma by targeting STAT3. Oncogene. (2017) 36:6627–39. doi: 10.1038/onc.2017.270 PMC5702716 28783167

[11] Wang YC Zheng LH Ma BA Zhou Y Zhang MH Zhang DZ. Clinical value of signal transducers and activators of transcription 3 (STAT3) gene expression in human osteosarcoma. Acta Histochem. (2011) 113:402–8. doi: 10.1016/j.acthis.2010.03.002 20546860

[12] Deng J Liu Y Lee H Herrmann A Zhang W Zhang C. S1PR1-STAT3 signaling is crucial for myeloid cell colonization at future metastatic sites. Cancer Cell. (2012) 21:642–54. doi: 10.1016/j.ccr.2012.03.039 PMC3360884 22624714

[13] Jing B Wang T Sun B Xu J Xu D Liao Y. IL6/STAT3 signaling orchestrates premetastatic niche formation and immunosuppressive traits in lung. Cancer Res. (2020) 80:784–97. doi: 10.1158/0008-5472.Can-19-2013 31848193

[14] Jones LM Broz ML Ranger JJ Ozcelik J Ahn R Zuo D. STAT3 establishes an immunosuppressive microenvironment during the early stages of breast carcinogenesis to promote tumor growth and metastasis. Cancer Res. (2016) 76:1416–28. doi: 10.1158/0008-5472.CAN-15-2770 PMC5052827 26719528

[15] Yu H Kortylewski M Pardoll D. Crosstalk between cancer and immune cells: role of STAT3 in the tumour microenvironment. Nat Rev Immunol. (2007) 7:41–51. doi: 10.1038/nri1995 17186030

[16] Yu H Pardoll D Jove R. STATs in cancer inflammation and immunity: A leading role for STAT3. Nat Rev Cancer. (2009) 9:798–809. doi: 10.1038/nrc2734 19851315 PMC4856025

[17] Ando K Mori K Corradini N Redini F Heymann D. Mifamurtide for the treatment of nonmetastatic osteosarcoma. Expert Opin Pharmacother. (2011) 12:285–92. doi: 10.1517/14656566.2011.543129 PMC3413631 21226638

[18] Meyers PA Schwartz CL Krailo MD Healey JH Bernstein ML Betcher D. Osteosarcoma: the addition of muramyl tripeptide to chemotherapy improves overall survival-a report from the children’s oncology group. J Clin Oncol. (2008) 26:633–8. doi: 10.1200/JCO.2008.14.0095 18235123

[19] Kleinerman ES Gano JB Johnston DA Benjamin RS Jaffe N. Efficacy of liposomal muramyl tripeptide (CGP 19835a) in the treatment of relapsed osteosarcoma. Am J Clin Oncol. (1995) 18:93–9. doi: 10.1097/00000421-199504000-00001 7900714

[20] Meyers PA Chou AJ. Muramyl tripeptide-phosphatidyl ethanolamine encapsulated in liposomes (L-MPT-PE) in the treatment of osteosarcoma. Curr Adv Osteosarcoma. (2014), 307–21. doi: 10.1007/978-3-319-04843-7_17 24924182

[21] Dancsok AR Gao D Lee AF Steigen SE Blay J-Y Thomas DM. Tumor-associated macrophages and macrophage-related immune checkpoint expression in sarcomas. Oncoimmunology. (2020) 9:1747340. doi: 10.1080/2162402X.2020.1747340 32313727 PMC7153829

[22] Wang Z Li B Li S Lin W Wang Z Wang S. Metabolic control of CD47 expression through lat2-mediated amino acid uptake promotes tumor immune evasion. Nat Commun. (2022) 13:6308. doi: 10.1038/s41467-022-34064-4 36274066 PMC9588779

[23] Gill J Gorlick R. Advancing therapy for osteosarcoma. Nat Rev Clin Oncol. (2021) 18:609–24. doi: 10.1038/s41571-021-00519-8 34131316

[24] van Duijn A van der Burg SH Scheeren FA. CD47/SIRPα Axis: bridging innate and adaptive immunity. J ImmunoTher Cancer. (2022) 10:e004589. doi: 10.1136/jitc-2022-004589 35831032 PMC9280883

[25] Deuse T Hu X Agbor-Enoh S Jang MK Alawi M Saygi C. The SIRPα-CD47 immune checkpoint in nk cells. J Exp Med. (2021) 218. doi: 10.1084/jem.20200839 PMC7802363 33416832

[26] Nath PR Pal-Nath D Mandal A Cam MC Schwartz AL Roberts DD. Natural killer cell recruitment and activation are regulated by CD47 expression in the tumor microenvironment. Cancer Immunol Res. (2019) 7:1547–61. doi: 10.1158/2326-6066.Cir-18-0367 PMC6726576 31362997

[27] Liu X Pu Y Cron K Deng L Kline J Frazier WA. CD47 blockade triggers T cell–mediated destruction of immunogenic tumors. Nat Med. (2015) 21:1209. doi: 10.1038/nm.3931 26322579 PMC4598283

[28] McCracken MN Cha AC Weissman IL. Molecular pathways: activating T cells after cancer cell phagocytosis from blockade of cd47 “Don’t eat me” Signals. Clin Cancer Res. (2015) 21:3597–601. doi: 10.1158/1078-0432.Ccr-14-2520 PMC4621226 26116271

[29] Groot Jd Ott M Wei J Kassab C Fang D Najem H. A first-in-human phase I trial of the oral P-STAT3 inhibitor WP1066 in patients with recurrent Malignant glioma. CNS Oncol. (2022) 11:CNS87. doi: 10.2217/cns-2022-0005 35575067 PMC9134932

[30] Madden T Kazerooni R Myer J Culotta K Donato N Johansen MJ. The preclinical pharmacology of WP1066, a potent small molecule inhibitor of the JAK2/STAT3 pathway. Cancer Res. (2006) 66:1139–40.

[31] Zielinski R Rusin A Madden T Conrad C Johansen M Fokt I. Development of orally bioavailable formulation of WP1066 and its evaluation *in vivo* . AACR. (2015) 75(15_supplement):4540. doi: 10.1158/1538-7445.AM2015-4540

[32] Hussain SF Kong L-Y Jordan J Conrad C Madden T Fokt I. A novel small molecule inhibitor of signal transducers and activators of transcription 3 reverses immune tolerance in malignant glioma patients. Cancer Res. (2007) 67:9630–6. doi: 10.1158/0008-5472.CAN-07-1243 17942891

[33] Kong L-Y Wu AS Doucette T Wei J Priebe W Fuller GN. Intratumoral mediated immunosuppression is prognostic in genetically engineered murine models of glioma and correlates to immunotherapeutic responses. Clin Cancer Res. (2010) 16:5722–33. doi: 10.1158/1078-0432.CCR-10-1693 PMC2999668 20921210

[34] Kong LY Wei J Sharma AK Barr J Abou-Ghazal MK Fokt I. A Novel Phosphorylated STAT3 Inhibitor Enhances T Cell Cytotoxicity against Melanoma through Inhibition of Regulatory T Cells. Cancer Immunol Immunother: CII. (2009) 58:1023–32. doi: 10.1007/s00262-008-0618-y PMC2674523 19002459

[35] Yang Y Peng Z Flores ER Kleinerman ES. Pramlintide: A novel therapeutic approach for osteosarcoma through metabolic reprogramming. Cancers (Basel). (2022) 14. doi: 10.3390/cancers14174310 PMC9454976 36077845

[36] Gross AC Cam H Phelps DA Saraf AJ Bid HK Cam M. Il-6 and CXCL8 mediate osteosarcoma-lung interactions critical to metastasis. JCI Insight. (2018) 3. doi: 10.1172/jci.insight.99791 PMC6141177 30135299

[37] Schmidt J Strauß GP Schön A Luz A Murray AB Melchiori A. Establishment and characterization of osteogenic cell lines from a spontaneous murine osteosarcoma. Differentiation. (1988) 39:151–60. doi: 10.1111/j.1432-0436.1988.tb00090.x 3243385

[38] Gordon N Koshkina NV Jia S-F Khanna C Mendoza A Worth LL. Corruption of the fas pathway delays the pulmonary clearance of murine osteosarcoma cells, enhances their metastatic potential, and reduces the effect of aerosol gemcitabine. Clin Cancer Res. (2007) 13:4503–10. doi: 10.1158/1078-0432.CCR-07-0313 PMC4503209 17671136

[39] Asai T Ueda T Itoh K Yoshioka K Aoki Y Mori S. Establishment and characterization of a murine osteosarcoma cell line (Lm8) with high metastatic potential to the lung. Int J Cancer. (1998) 76:418–22. doi: 10.1002/(SICI)1097-0215(19980504)76:3<418::AID-IJC21>3.0.CO;2-5 9579581

[40] Kim SY Lee CH Midura BV Yeung C Mendoza A Hong SH. Inhibition of the CXCR4/CXCL12 chemokine pathway reduces the development of murine pulmonary metastases. Clin Exp Metastasis. (2008) 25:201–11. doi: 10.1007/s10585-007-9133-3 PMC2730112 18071913

[41] Fan TM Roberts RD Lizardo MM. Understanding and modeling metastasis biology to improve therapeutic strategies for combating osteosarcoma progression. Front Oncol. (2020) 10:13. doi: 10.3389/fonc.2020.00013 32082995 PMC7006476

[42] Ott M Kassab C Marisetty A Hashimoto Y Wei J Zamler D. Radiation with stat3 blockade triggers dendritic cell–T cell interactions in the glioma microenvironment and therapeutic efficacy. Clin Cancer Res. (2020) 26:4983–94. doi: 10.1158/1078-0432.CCR-19-4092 PMC9341321 32605912

[43] Kong L-Y Abou-Ghazal MK Wei J Chakraborty A Sun W Qiao W. A novel inhibitor of STAT3 activation is efficacious against established central nervous system melanoma and inhibits regulatory T cells. Clin Cancer Res. (2008) 14:5759. doi: 10.1158/1078-0432.CCR-08-0377 18794085 PMC2583362

[44] Willingham SB Volkmer J-P Gentles AJ Sahoo D Dalerba P Mitra SS. The CD47-signal regulatory protein alpha (Sirpa) interaction is a therapeutic target for human solid tumors. Proc. Natl. Acad. Sci. (PNAS). (2012) 109:6662–7. doi: 10.1073/pnas.1121623109 PMC3340046 22451913

[45] Ager CR Boda A Rajapakshe K Lea ST Di Francesco ME Jayaprakash P. High potency sting agonists engage unique myeloid pathways to reverse pancreatic cancer immune privilege. J ImmunoTher Cancer. (2021) 9:e003246. doi: 10.1136/jitc-2021-003246 34341132 PMC8330562

[46] Headen DM Woodward KB Coronel MM Shrestha P Weaver JD Zhao H. Local immunomodulation with fas ligand-engineered biomaterials achieves allogeneic islet graft acceptance. Nat Mater. (2018) 17:1. doi: 10.1038/s41563-018-0099-0 PMC6060019 29867165

[47] Vonderheide RH. CD47 blockade as another immune checkpoint therapy for cancer. Nat Med. (2015) 21:1122–3. doi: 10.1038/nm.3965 26444633

[48] Bitsch R Kurzay A Kurt FÖ Torre CDL Lasser S Lepper A. STAT3 inhibitor napabucasin abrogates mdsc immunosuppressive capacity and prolongs survival of melanoma-bearing mice. J ImmunoTher Cancer. (2022) 10:e004384. doi: 10.1136/jitc-2021-004384 35301236 PMC8932276

[49] Zhang M Meng Y Ying Y Zhou P Zhang S Fang Y. Selective activation of STAT3 and STAT5 dictates the fate of myeloid progenitor cells. Cell Death Discov. (2023) 9:274. doi: 10.1038/s41420-023-01575-y 37507383 PMC10382539

[50] Sica A Mantovani A. Macrophage plasticity and polarization: *in vivo* veritas. J Clin Invest. (2012) 122:787–95. doi: 10.1172/JCI59643 PMC3287223 22378047

[51] Mohanty S Aghighi M Yerneni K Theruvath JL Daldrup-Link HE. Improving the efficacy of osteosarcoma therapy: combining drugs that turn cancer cell ‘Don’t eat me’signals off and ‘Eat me’signals on. Mol Oncol. (2019) 13:2049–61. doi: 10.1002/1878-0261.12556 PMC6763764 31376208

[52] Marina NM Smeland S Bielack SS Bernstein M Jovic G Krailo MD. Comparison of mapie versus map in patients with a poor response to preoperative chemotherapy for newly diagnosed high-grade osteosarcoma (Euramos-1): an open-label, international, randomised controlled trial. Lancet Oncol. (2016) 17:1396–408. doi: 10.1016/s1470-2045(16)30214-5 PMC5052459 27569442

[53] Lagmay JP Krailo MD Dang H Kim A Hawkins DS Beaty O 3rd. Outcome of patients with recurrent osteosarcoma enrolled in seven phase ii trials through children’s cancer group, pediatric oncology group, and children’s oncology group: learning from the past to move forward. J Clin Oncol. (2016) 34:3031–8. doi: 10.1200/jco.2015.65.5381 PMC5012712 27400942

[54] Tawbi HA Burgess M Bolejack V Van Tine BA Schuetze SM Hu J. Pembrolizumab in advanced soft-tissue sarcoma and bone sarcoma (Sarc028): A multicentre, two-cohort, single-arm, open-label, phase 2 trial. Lancet Oncol. (2017) 18:1493–501. doi: 10.1016/S1470-2045(17)30624-1 PMC7939029 28988646

[55] Ligon JA Choi W Cojocaru G Fu W Hsiue EH-C Oke TF. Pathways of immune exclusion in metastatic osteosarcoma are associated with inferior patient outcomes. J ImmunoTher Cancer. (2021) 9:e001772. doi: 10.1136/jitc-2020-001772 34021032 PMC8144029

[56] Buddingh EP Kuijjer ML Duim RA Bürger H Agelopoulos K Myklebost O. Tumor-infiltrating macrophages are associated with metastasis suppression in high-grade osteosarcoma: A rationale for treatment with macrophage activating agents. Clin Cancer Res. (2011) 17:2110–9. doi: 10.1158/1078-0432.CCR-10-2047 21372215

[57] Xiao Q Zhang X Wu Y Yang Y. Inhibition of macrophage polarization prohibits growth of human osteosarcoma. Tumor Biol. (2014) 35:7611–6. doi: 10.1007/s13277-014-2005-y 24798973

[58] Theruvath J Menard M Smith BAH Linde MH Coles GL Dalton GN. Anti-gd2 synergizes with cd47 blockade to mediate tumor eradication. Nat Med. (2022) 28:333–44. doi: 10.1038/s41591-021-01625-x PMC9098186 35027753

[59] Wendt MK Balanis N Carlin CR Schiemann WP. STAT3 and epithelial-mesenchymal transitions in carcinomas. Jakstat. (2014) 3:e28975. doi: 10.4161/jkst.28975 24843831 PMC4024059

[60] Zhao C Li H Lin H-J Yang S Lin J Liang G. Feedback activation of STAT3 as a cancer drug-resistance mechanism. Trends Pharmacol Sci. (2016) 37:47–61. doi: 10.1016/j.tips.2015.10.001 26576830

[61] Horiguchi A Asano T Kuroda K Sato A Asakuma J Ito K. STAT3 inhibitor WP1066 as a novel therapeutic agent for renal cell carcinoma. Br J Cancer. (2010) 102:1592–9. doi: 10.1038/sj.bjc.6605691 PMC2883159 20461084

[62] Tsujita Y Horiguchi A Tasaki S Isono M Asano T Ito K. STAT3 inhibition by WP1066 suppresses the growth and invasiveness of bladder cancer cells. Oncol Rep. (2017) 38:2197–204. doi: 10.3892/or.2017.5902 28849140

[63] Ferrajoli A Faderl S Van Q Koch P Harris D Liu Z. WP1066 disrupts Janus kinase-2 and induces caspase-dependent apoptosis in acute myelogenous leukemia cells. Cancer Res. (2007) 67:11291–9. doi: 10.1158/0008-5472.Can-07-0593 18056455

[64] Chao MP Alizadeh AA Tang C Myklebust JH Varghese B Gill S. Anti-CD47 antibody synergizes with rituximab to promote phagocytosis and eradicate non-hodgkin lymphoma. Cell. (2010) 142:699–713. doi: 10.1016/j.cell.2010.07.044 20813259 PMC2943345

[65] Chao MP Jaiswal S Weissman-Tsukamoto R Alizadeh AA Gentles AJ Volkmer J. Calreticulin is the dominant pro-phagocytic signal on multiple human cancers and is counterbalanced by CD47. Sci Trans Med. (2010) 2:63ra94–4. doi: 10.1126/scitranslmed.3001375 PMC4126904 21178137

[66] Mohanty S Yerneni K Theruvath JL Graef CM Nejadnik H Lenkov O. Nanoparticle enhanced mri can monitor macrophage response to CD47 mab immunotherapy in osteosarcoma. Cell Death Dis. (2019) 10:36. doi: 10.1038/s41419-018-1285-3 30674867 PMC6367456

[67] ClinicalTrials.gov. WP1066 in Children with Refractory and Progressive or Recurrent Malignant Brain Tumors (2023). Available online at: https://clinicaltrials.gov/study/NCT04334863 (Accessed April 28, 2025).

[68] Onclive.com. Fda Grants 3 Rare Pediatric Disease Designations to STAT3 Inhibitor WP1066 (2021). Available online at: https://www.onclive.com/view/fda-grants-3-rare-pediatric-disease-designations-to-stat3-inhibitor-wp1066 (Accessed April 28, 2025).

[69] Yang H Xun Y You H. The landscape overview of CD47-based immunotherapy for hematological Malignancies. Biomarker Res. (2023) 11:15. doi: 10.1186/s40364-023-00456-x PMC9893585 36726125

[70] Serani S. Fda Halts Clinical Studies of Magrolimab in Aml, Mds (2024). Available online at: https://www.targetedonc.com/view/fda-halts-clinical-studies-of-magrolimab-in-aml-mds (Accessed April 28, 2025).

