# Supplementary material for: STAT3 inhibition in combination with CD47 blockade inhibits osteosarcoma lung metastasis
Source: Front Immunol. 2025 Jun 3;16:1608375. doi: 10.3389/fimmu.2025.1608375 (PMC12170595; doi:10.3389/fimmu.2025.1608375)
Supplement: Supplementary file 1 [file DataSheet1.pdf]

Supplementary Information

Supplementary materials and methods

Cell proliferation and resazurin assay

K7M3, DLM8, OS17 and LM7 cells were seeded at density of 5000 cell/ well in 96-well culture plates overnight followed by treating with different indicated concentration of WP1066. Plates were incubated at 37 °C and each well was imaged at 3 hrs intervals using IncuCyte S3 live-cell imaging systems for 72 hrs and followed with resazurin assay. Each image was analyzed for cell confluency.

Resazurin assay was performed as described before (41). Briefly, after 72 hrs of cell culture with WP1066, cells were treated with resazurin viability reagent and incubated for additional 2 hrs at 37 °C. Resorufin fluorescence was measured at 545 nm excitation and 595 nm emission using (CLARIOPstarplus). Cell viability was normalized to blank wells containing media only.

Western blot

Cell lysate was prepared using RIPA buffer (Thermofisher) with protease and phosphatase inhibitors (Thermofisher). Protein separation was done in SDS-PAGE followed by transferring onto PVDF membrane. The membrane was probed with primary antibody against pSTAT3Y705 (cell signaling), tSTAT3 (cell signaling), or beta-actin (Sigma), followed by HRP-conjugated secondary antibody and visualized with Pierce ECL Western Blotting substrate (Thermofisher). Blot were analyzed using Fiji (ImageJ) software.

Caspase-3/7 activation and Annexin V assay

Caspase-3/7 activation and expression of Annexin V upon treatment with WP1066 was assessed with Incucyte S3 system following manufacturer's protocol. Briefly, 5000 cells/well were seeded on 96 well plate. After overnight culture, cells were treated with WP1066 at indicated concentration or DMSO. Caspase-3/7 dye (Sartorius) was added to each well at final concentration of 5 µM. To detect the expression of phosphatidyl serine (PS) on surface of apoptotic cell by Annexin V assay, Incucyte Annexin V Green dye (Sartorius) was used. Each well was imaged for GFP signals every 3 hrs for 72 hrs in IncuCyte S3 system.

Phagocytosis assay

Phagocytosis assay was performed as described before (43). K7M3 cells were treated with WP1066 or DMSO for 24 hrs. Target cells were harvested and labelled with CFSE (2.5 µM) followed by co-culturing with LPS treated (10 ng/ml, 24 hrs) RAW264.7 cells in ultra-low attachment plates at a ratio of 2:1. After 4 hrs of co-culturing, cells were harvested and analyzed for phagocytosis percentage of CD11b+F480+ CFSE+ macrophage by flow cytometry.

Supplementary Fig. S1

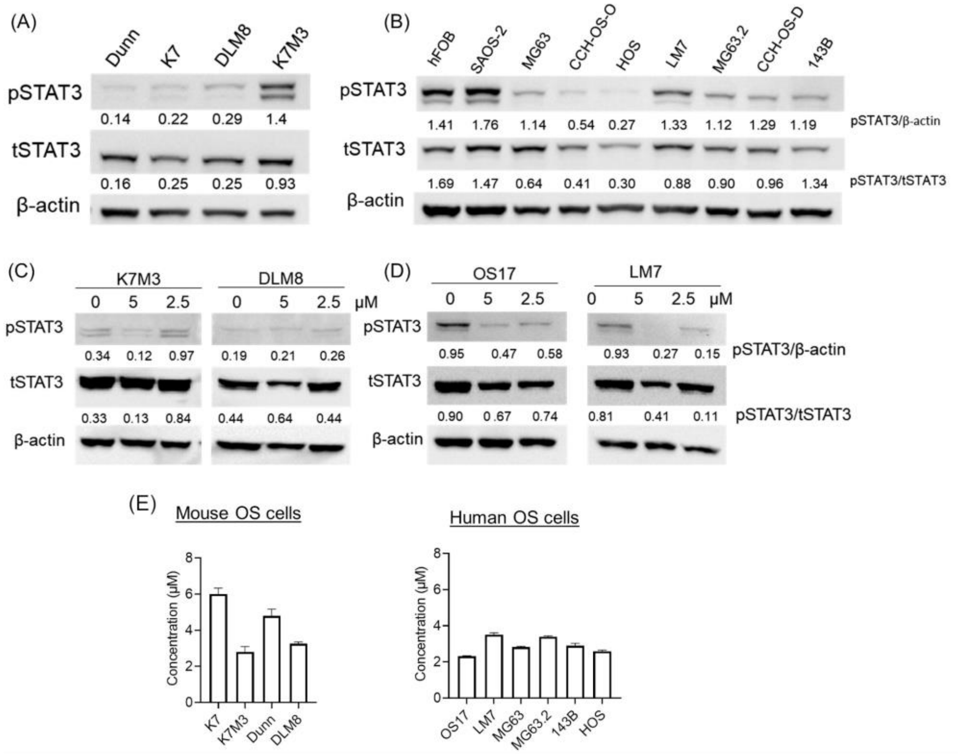

Supplementary Fig. S1. WP1066 inhibits activation of STAT3 in OS cell lines. (A, B) Expression of pSTAT3, tSTAT3 and β-actin in mouse and human OS cell lines. Immunoblot analysis of cell lysates from mouse OS cell lines (A) and human OS and normal osteoblast cell lines (B) with antibodies specific for pSTAT3^{Y705}, total STAT3 and β-actin as the internal control. (C, D) WP1066 inhibits activation of STAT3^{Y705}. Mouse (C) and human (D) OS cells were incubated with WP1066 at different concentration for 24 hrs prior to immunoblotting analysis. Relative protein expression values of pSTAT3^{Y705} are shown by normalizing values to tSTAT3 and β-actin in A-D. (E) IC50 values for WP1066 against multiple mouse and human OS cells. Mouse and human OS cells were treated with different concentration of WP1066 for 72 hrs followed by resazurin assay to determine IC50 values.

Supplementary Fig. S2

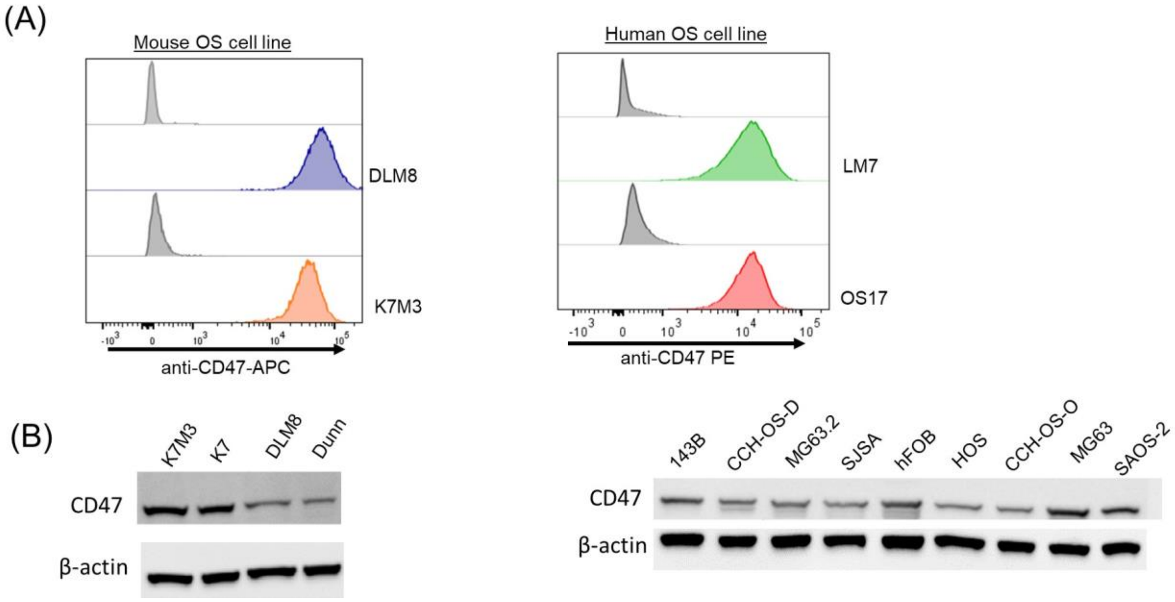

Supplementary Fig. S2. Expression of CD47 in OS cells. CD47 expression in mouse and human OS cells as measured by flow cytometry (A) and immunoblot analysis (B).

Supplementary Fig. S3

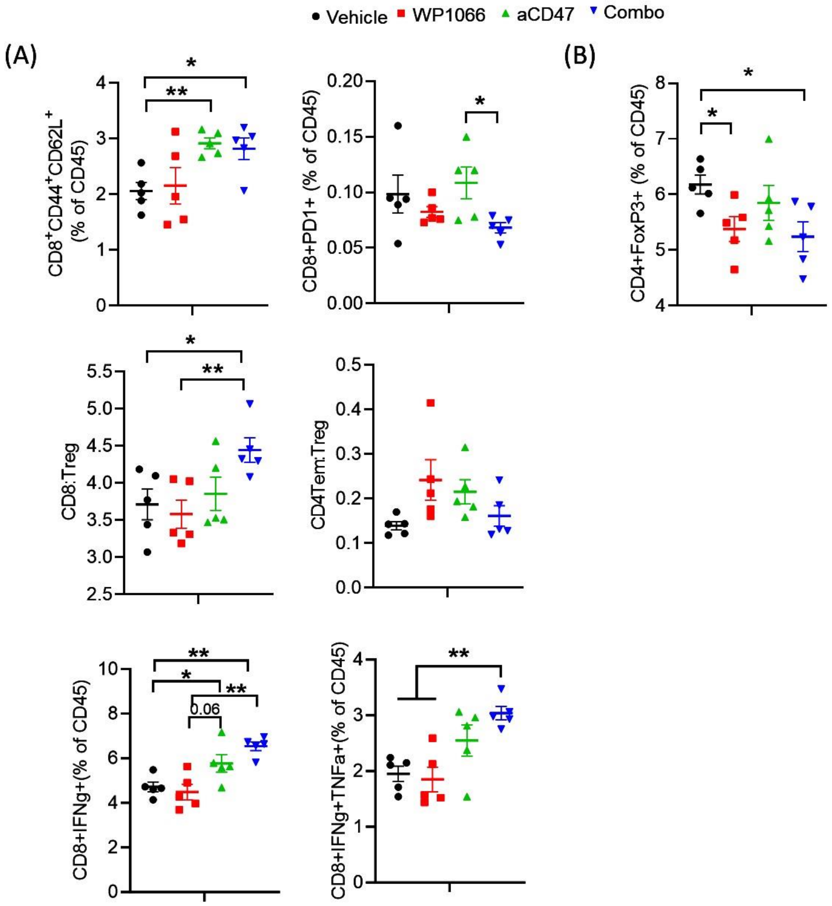

Supplementary Fig. S3. Flow cytometric analysis of immune cells in LDLNs.

Single cells from LDLNs were analyzed for (A) CD8Tcm ($CD8^{+}CD44^{+}CD62L^{+}$), $CD8^{+}PD1^{+}$ and cytotoxic IFN-γ/TNF-α secreting CD8 T cells and (B) FoxP3 expressing Tregs ($CD4^{+}FoxP3^{+}$). Statistical significance calculated by Mann-Whitney Test. *p<0.05, **p<0.01

Supplementary Table 1. Fluorochrome conjugated antibodies for flow cytometric analysis.

Marker	Clone	Company	Cat#
CD45	30-F11	Biolegend	103116
CD11b	M1/70	Biolegend	101228
F4/80	BM8	Biolegend	123130
CD11c	N418	Biolegend	117310
CD80	16-10A1	Biolegend	104729
MHC-II (I-A/I-E)	M5/114.15.2	Biolegend	107608
Ly6C	HK1.4	Biolegend	128022
Ly6G	1A8	Biolegend	127618
CD27	LG.3A10	Biolegend	124208
PD1	29F.1A12	Biolegend	135220
CD8a	53-6.7	Biolegend	100712
CD3	17A2	Biolegend	100228
CD4	GK1.5	Biolegend	100423
CD44	IM7	Biolegend	103026
CD62L	MEL-14	Biolegend	104408
CD49b	DX5	Biolegend	108908
FoxP3	FJK-16s	Invitrogen	45-5773-82
PDL1	10F.9G2	Biolegend	124308
IFN-g	XMG1.2	Biolegend	505826
TNF-a	MP6-XT22	Biolegend	506322
